# Supplementary material for: Hydrogel Formulations of Antibacterial Pyrazoles Using a Synthesized Polystyrene-Based Cationic Resin as a Gelling Agent
Source: Int J Mol Sci. 2023 Jan 6;24(2):1109. doi: 10.3390/ijms24021109 (PMC9862678; doi:10.3390/ijms24021109)
Supplement: Supplementary file 1 [file ijms-24-01109-s001.zip › ijms-2134239-supplementary.pdf]

International Journal of
Molecular Sciences

MDPI

Supplementary Materials

Hydrogel Formulations of Antibacterial Pyrazoles Using a Synthesized Polystyrene-Based Cationic Resin as Gelling Agent

Silvana Alfei [1,*], Guendalina Zuccari [1,*], Eleonora Russo [2], Carla Villa [2], and Chiara Brullo [2]

[1] Department of Pharmacy, Section of chemistry and pharmaceutical and food technologies, University of Genoa, Viale Cembrano, 4, 16148 Genoa, Italy
[2] Department of Pharmacy (DIFAR), Section of Medicinal Chemistry and Cosmetic Product, University of Genoa, Viale Benedetto XV, 3, 16132 Genoa, Italy
* Correspondence: alfei@difar.unige.it (S.A.); zuccari@difar.unige.it (G.Z.); Tel.: +39-010-355-2296 (S.A.)

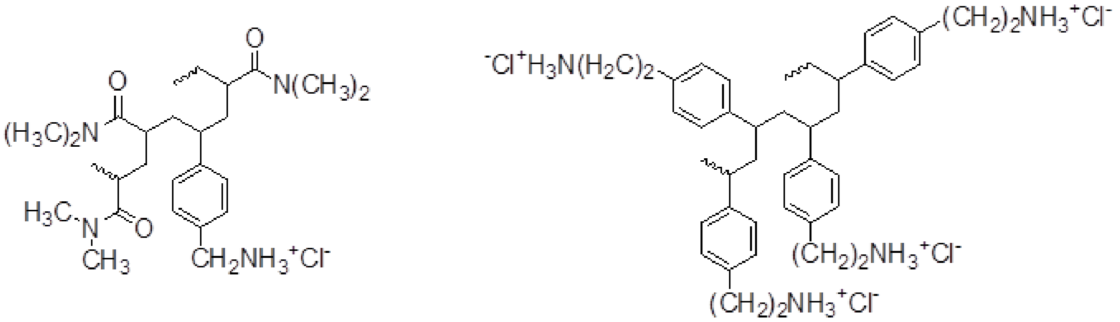

CP1 OP2

Figure S1. Structure of copolymer CP1 (left) and homopolymer OP2 (right).

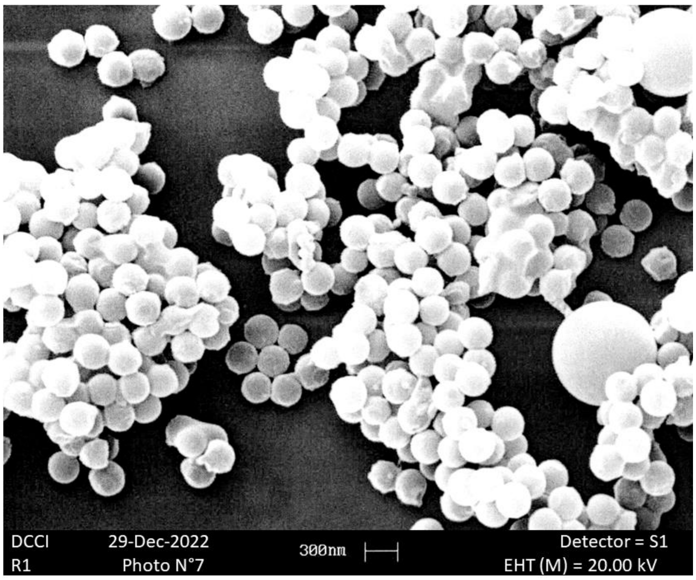

Figure S2. SEM image of resin R1.

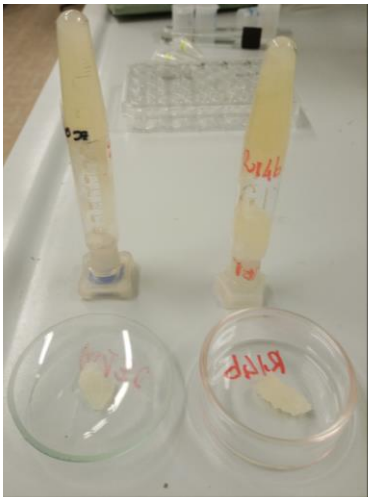

Figure S3. Appearance of R1HG-3c (left side) and R1HG-4b (right side) at their equilibrium degree of swelling (EDS).

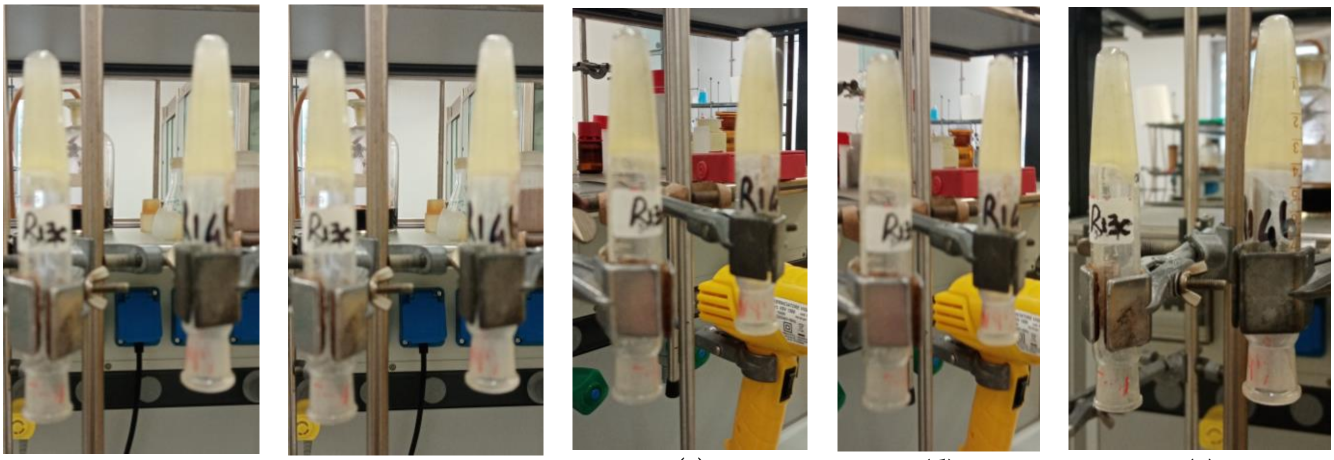

(a) **(b)** **(c)** **(d)** **(e)**

Figure S4. Appearance of 3 mL of R1HG-3c (left side of the images) and 4 mL of R1HG-4b (right side of the images) in the inverted position just prepared (**a**), after 1 (**b**), 2 (**c**), 3 (**d**) and 4 months (**e**) from first preparation staying at room temperature.

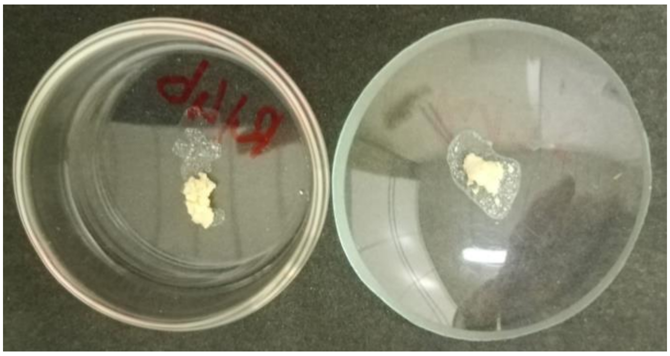

Figure S5. Appearance of fully dried D-R1HG-3c (right side) and D-R1HG-4b (left side) when a constant weight was reached.

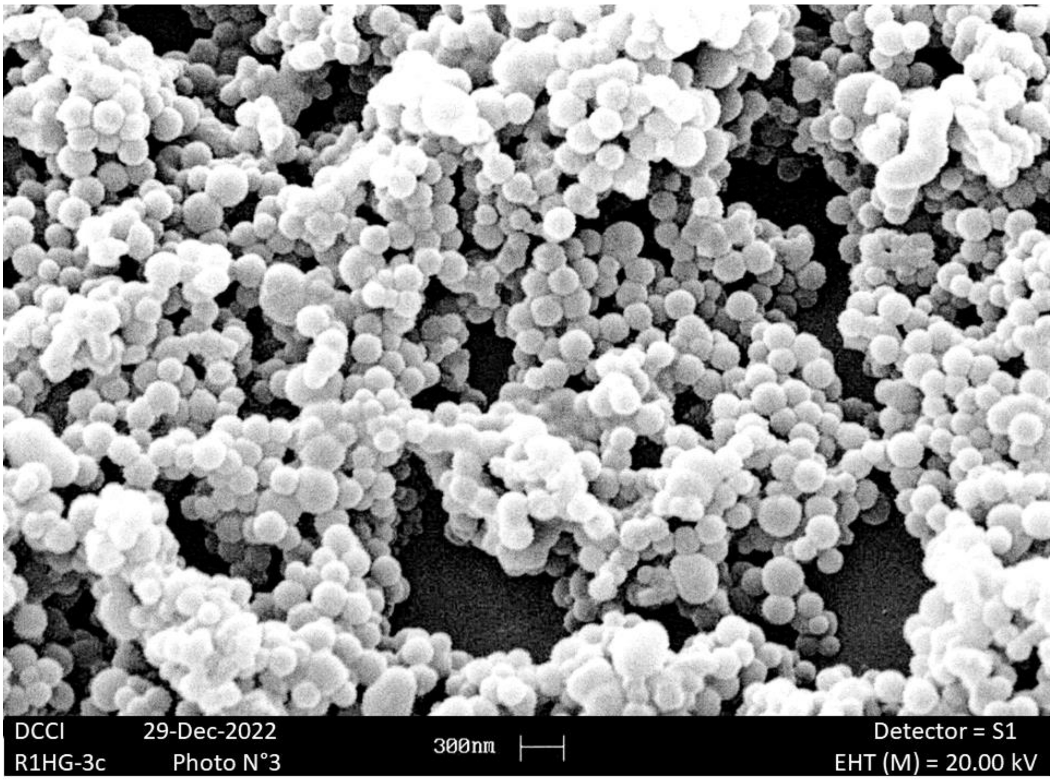

Figure S6. SEM image of the dried D-R1HG-3c.

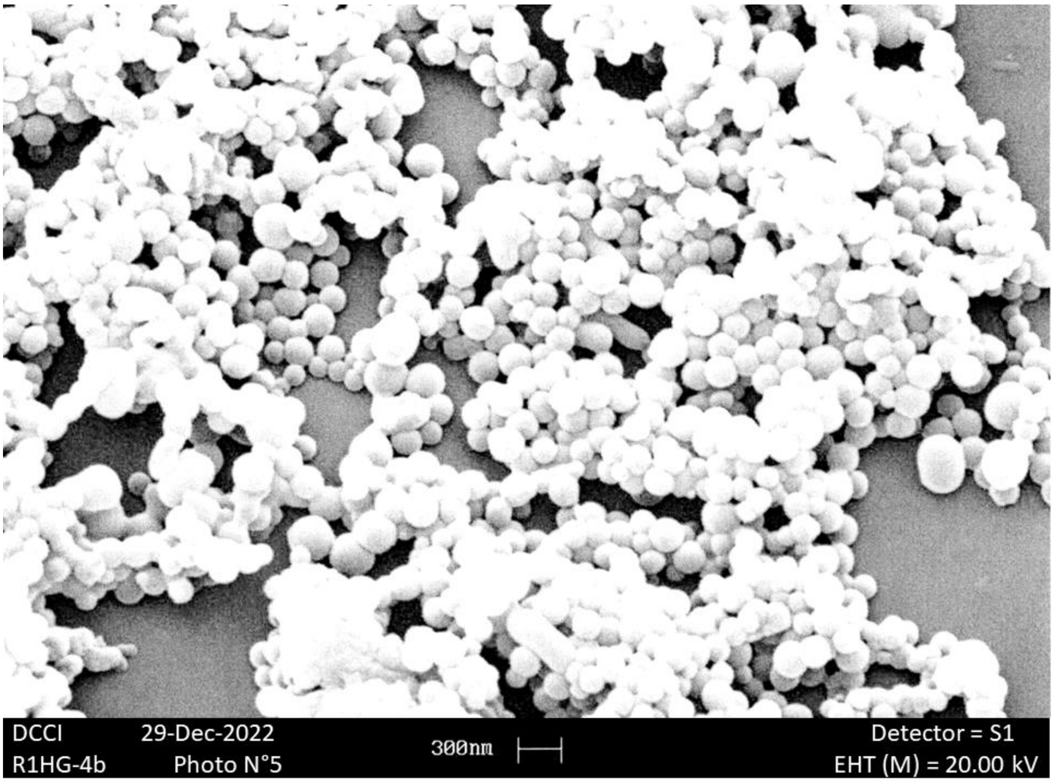

Figure S7. SEM image of the dried D-R1HG-4b.
